# A 55-Year-Old Male With Systemic Gout Complicated by Septic Shock

**DOI:** 10.7759/cureus.40764

**Published:** 2023-06-21

**Authors:** Jay E Garza, Quoc-Bao Nguyen, Daniel V Wang

**Affiliations:** 1 Dermatology, McGovern Medical School, Houston, USA; 2 Hospital Medicine, Memorial Hermann Texas Medical Center, Houston, USA

**Keywords:** gout flare, gram positive bacteremia, chronic ulcer, mssa bacteremia, severe sepsis, tophaceous gout

## Abstract

Tophaceous gout is the systemic deposition of uric acid which can induce cutaneous ulceration. We present the case of a 55-year-old male with chronic tophaceous gout whose initial presentation was complicated by septic shock due to methicillin-sensitive *Streptococcus aureus* bacteremia and superinfection of many of his affected joints. The case and discussion will focus on the extent of his infections and approaches to preventative care.

## Introduction

Tophaceous gout is the deposition of crystalline uric acid (UA) in the tissues. Most cases rarely develop such extensive involvement on account of urate-lowering therapies (ULT) [1]. However, when the UA concentration supersaturates the extracellular fluid, monosodium urate (MSU) can precipitate and deposit within joints, tendons, ligaments, and subcutaneous tissue as tophi [2], which promotes a systemic, pro-inflammatory state [3].

Chronic inflammation and large tophi can cause skin breakdown, joint dysfunction, and open sores that leave a patient susceptible to infectious complications. In a study of over 430,000 patients, patients with gout were more likely to develop septic arthritis with a multivariate-adjusted hazard ratio of 2.6 [4].

## Case presentation

**Figure 1:**
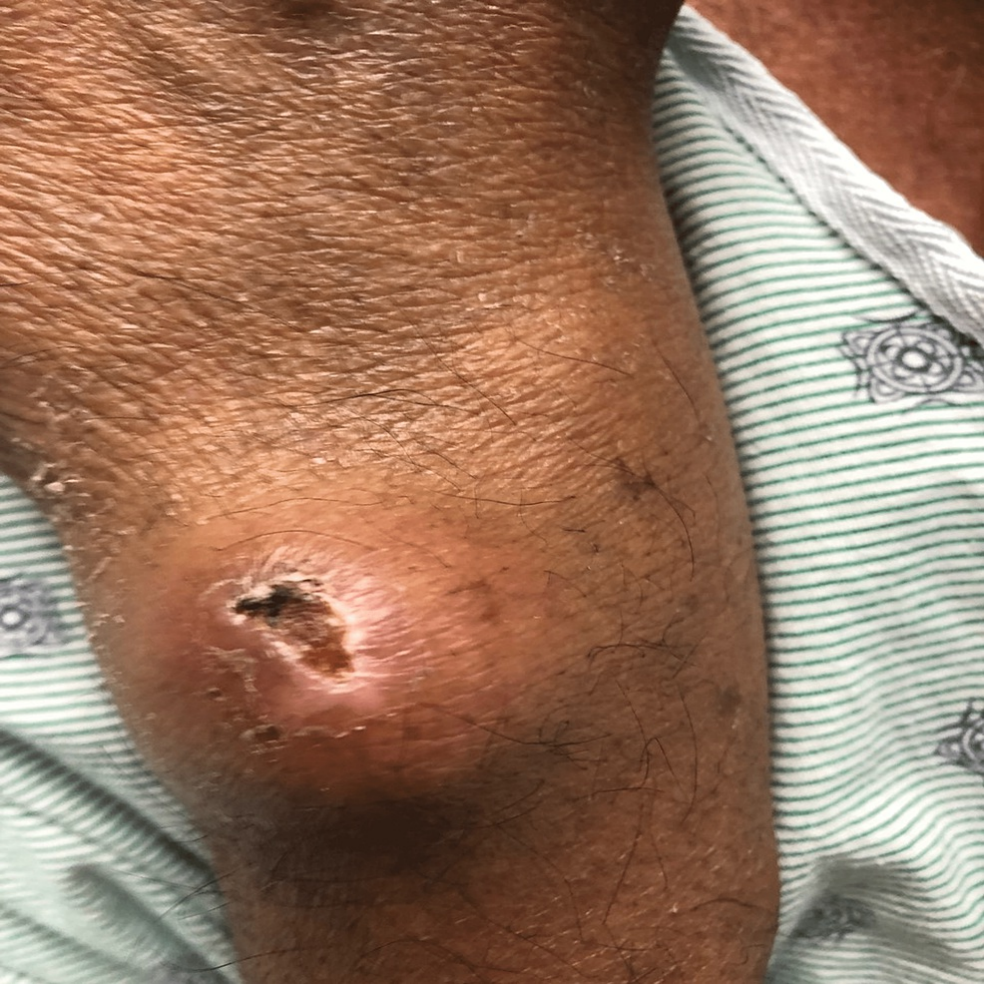
Right forearm tophus and ulceration.

**Figure 2:**
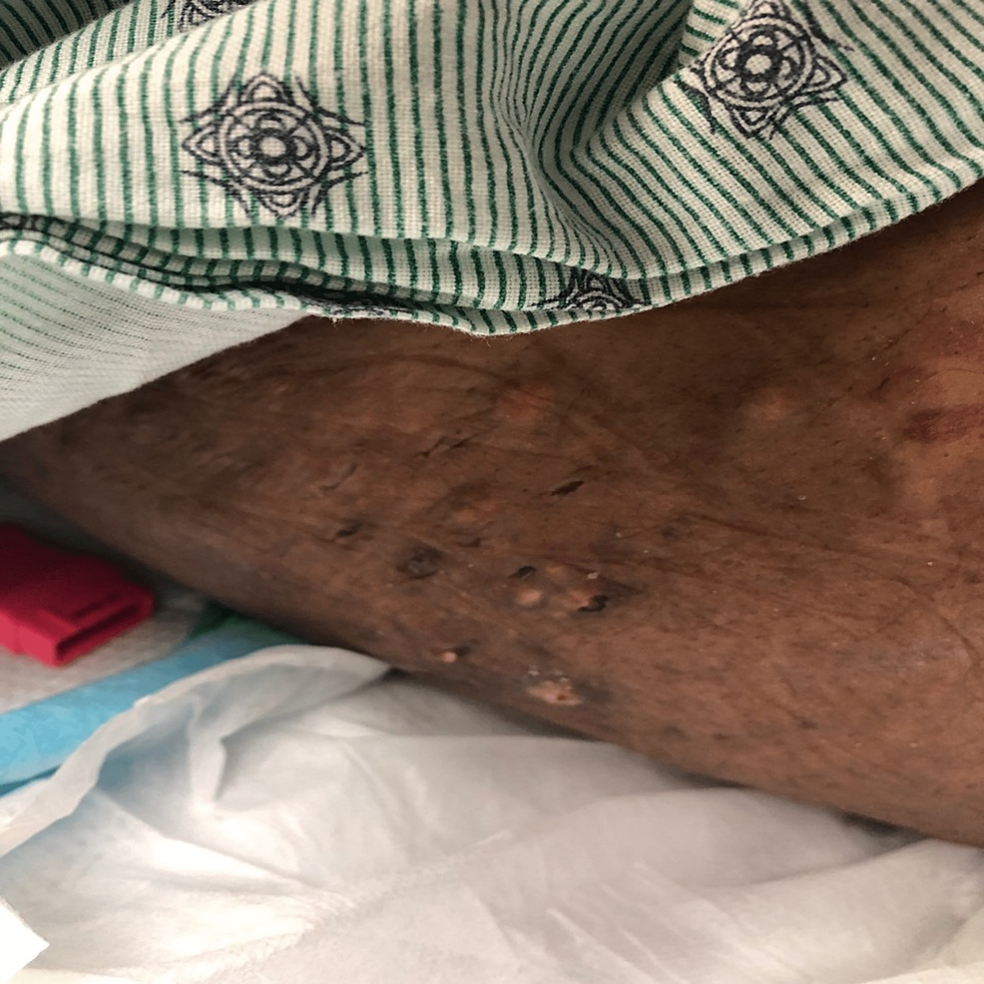
Ulceration and tophaceous involvement of the left flank.

**Figure 3:**
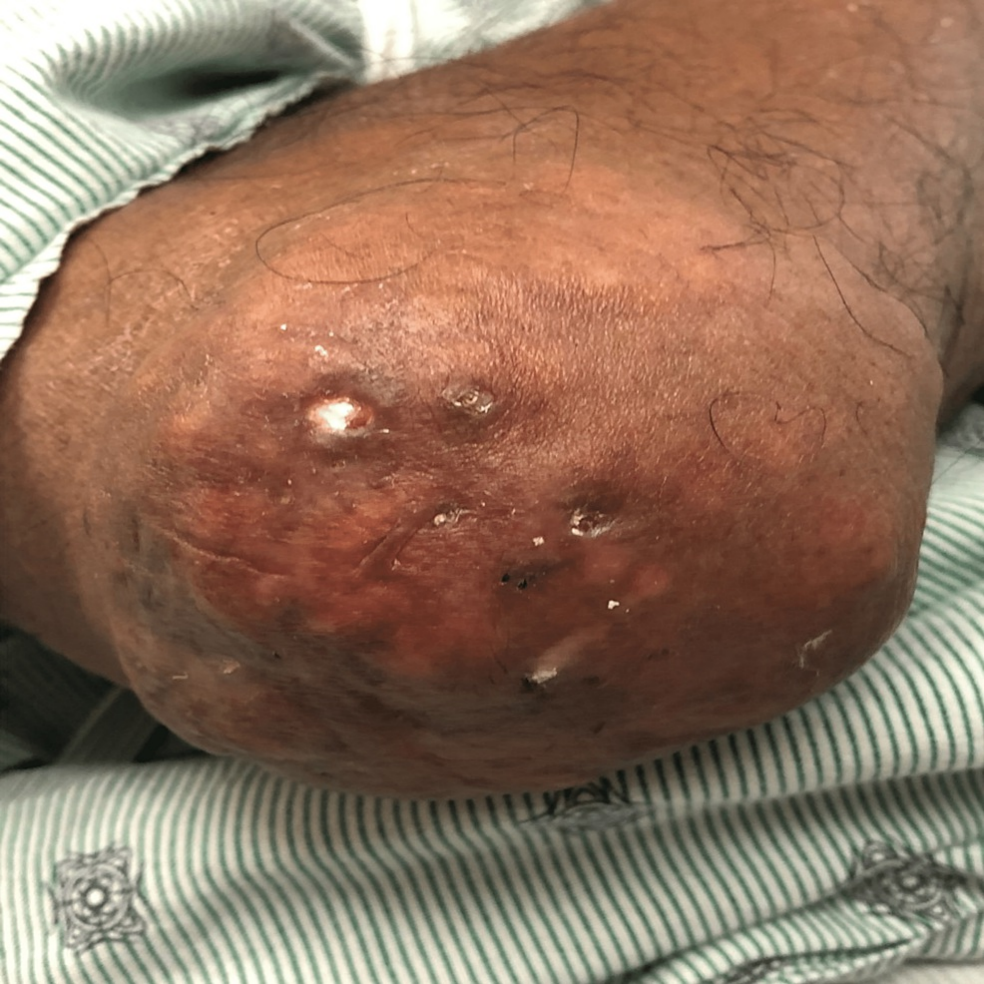
Left elbow tophus.

A 55-year-old male with a past medical history of tophaceous gout presented to the hospital with diffuse joint pain and swelling. On examination, there were multiple gouty tophi involving the hands, arms (Figure 1), elbows (Figure 2), right shoulder, knees, feet, ankles, and subcutaneous tissues of his flank (Figure 3). His vitals (Table 1) indicated that he was septic according to systemic inflammatory response syndrome signs [5]. The patient was immediately started on clindamycin 600 mg once daily and vancomycin twice daily. Cefazolin 2 g three times daily was started as his blood culture tested positive for methicillin-sensitive *Streptococcus aureus* (MSSA). The patient’s family noted that he had inconsistently been taking febuxostat for his gout control. The patient was also started on high-dose steroids of 40 mg methylprednisolone twice daily. The patient remained on that regimen for 12 days until he began to develop incontinence and saddle paresthesia.

**Table 1:** Patient vitals and lab values for SIRS criteria. Four of the four SIRS criteria are met [5]. SIRS = systemic inflammatory response syndrome

Vital signs and labs (units)	Value	Reference
Heart rate (beats/minute)	102	60–100
Respiratory rate (breaths/minute)	30	12–20
White blood cells (10^3^/µL)	16.2	3.7–10.4
Temperature (°F)	98.4	96.4–99

He was transferred to another hospital for a neurosurgical consult for concern for compression where his steroids were stopped, and his antibiotics were changed to ceftaroline 600 mg twice daily and daptomycin 700 mg daily. Colchicine 0.6 mg daily along with allopurinol 600 mg daily were added for his gout. Ultrasound and CT did not find localized fluid within the spine, and outpatient follow-up was discussed with the patient for inflammation within the spine. Imaging of the shoulder showed an abscess which was drained shortly thereafter. The antibiotic regimen was increased to 600 mg ceftaroline three times daily, 900 mg daptomycin daily, and clindamycin 900 mg three times daily, and rifampin 600 mg daily was added for continuing fevers and lack of source control. The patient’s mental and hemodynamic status continued to worsen, and he was intubated to protect his airway. Rifampin and ceftaroline were held intermittently for drops in platelets below 50 K/µL. Despite consistent and appropriate antibiotic coverage, the patient had stayed in a persistent shock state, and ciprofloxacin 400 mg three times daily and micafungin 100 mg daily were added to his regimen. The ciprofloxacin was quickly removed after three days as there was no evidence from blood cultures or hepatobiliary iminodiacetic acid (HIDA) scans to support its use. The antibiotic regimen was changed shortly thereafter to cover for a possible intra-abdominal process despite a negative HIDA scan because appropriate MSSA coverage was not improving the patient’s condition. His antibiotics were switched to vancomycin 1.5 g daily, meropenem 2 g three times daily, rifampin 600 mg daily, and daptomycin 900 mg daily were continued.

The patient began experiencing episodic oliguric acute kidney injury (AKI) and the vancomycin was changed to renally dosed. His creatinine at admission was 0.52 mg/dL and had increased to 1.47 mg/dL. His kidney failure warranted continuous renal replacement therapy and a dosage adjustment of vancomycin to renally dosed, meropenem to 500 mg daily, and daptomycin to every 48 hours. The patient’s allopurinol was also changed to a renal dosage of 200 mg daily. Once moved from continuous renal replacement therapy to hemodialysis three times per week, his allopurinol was adjusted to 200 mg three times per week and colchicine 0.3 mg three times per week.

The sources of infection were determined to be the right total knee arthroplasty and the multiple gouty tophi. However, due to hemodynamic instability, the decision to not excise the tophi was made. Physiologic hydrocortisone was started as hemodynamic instability was in part due to adrenal insufficiency after an AM cortisol was reported at 3.6 µg/dL. Daptomycin was switched to ceftaroline 600 mg three times daily to provide broader pulmonary coverage as there was a concern for hospital-acquired pneumonia. The ceftaroline was removed after five days of treatment. With the resolution of the AKI and removal of hemodialysis, meropenem was increased to 2 g daily, and rifampin and vancomycin were continued at 600 mg daily and renal dosage, respectively. The allopurinol was removed, and colchicine was given 0.3 mg every 48 hours.

The patient’s eosinophils rose periodically throughout the treatment course but were now consistently high warranting discontinuation of rifampin. With the improvement of the patient’s hemodynamic and neurologic status, the patient was extubated, and meropenem was stopped shortly thereafter, followed by the stoppage of micafungin and vancomycin. The patient’s eosinophilia improved with the cessation of the other antibiotics and the administration of physiologic hydrocortisone for adrenal insufficiency. Oral doxycycline 100 mg was given twice daily to the patient for indefinite suppression. The patient’s physiologic and neurologic status had improved and he was able to work with physical and occupational therapy. The patient was discharged with doxycycline and allopurinol with no recurrence of flares for the following 12 months.

## Discussion

Our case displays a complicated course of inadequately treated tophaceous gout. The concomitant bacteremia made the determination of the source of infection particularly difficult. While the tophi and the abscesses were identified as sources, the antibiotics administered seemed to be ineffective even after drainage of the abscesses. In this case, the tophi could not be excised due to the patient’s hemodynamic instability further skewing our perception of what could be causing the bacteremia. The patient had only one positive blood culture at the beginning of treatment, so other sources of infection were challenging to rule out which further broadened our antibiotic regimen.

The overall prevalence of gout among US adults (>20 years old) was 3.9%, corresponding to a total affected population of 9.2 million in 2016 [1]. However, the age-adjusted prevalence of gout and hyperuricemia has remained unchanged since 2008 [1]. In the United States, gout is found three times more often in men than women [6]. Comorbidities such as hypertension are present in up to three-quarters of gout patients, and chronic kidney disease of stage 3 or greater severity is present in many patients with gout. Both of these can be in the causal pathway of its association with cardiovascular disease and stroke [6].

Current chronic gout management includes the use of ULTs such as allopurinol, pegloticase, and febuxostat. A sustained and significant reduction in serum UA (<0.3 mmol/L) can lead to tophi reduction regardless of which medication is used [7]. Acute flare management is particularly challenging because many patients who suffer from gout have comorbid conditions such as chronic kidney disease, heart failure, diabetes, or hypertension. This leaves traditional medications such as colchicine, non-steroidal anti-inflammatory drugs, and steroids particularly worrisome and often avoided in acute flares [8]. Anakinra, an interleukin 1-receptor antagonist, has been introduced as an effective acute gout flare treatment in patients, especially those with multiple comorbidities [8].

While intensive wound management and appropriate preventative ULTs can be provided in the hospital setting, the continuation of such care at home is essential in a patient’s longitudinal care [7,9]. The management of acute flares now has new pharmacologic agents such as Anakinra that do not interfere with comorbidities but may offer trepidation to clinicians when the patient has a concomitant infection [8,10]. Further investigation into novel pharmacologic treatments that do not interfere with comorbidities on whether they exacerbate concomitant infection may be warranted [10]. Therefore, caregiver education and training in wound care and preventative ULTs are of particular importance.

## Conclusions

This case shows a complicated course of inadequately treated tophaceous gout. The tophi and subsequent abscesses while obvious could have obscured other sources of infection. Thus, a long course of broad-regimen antibiotics was necessary to treat the bacteremia. Lack of access to pharmaceuticals that can treat gout without interfering with comorbidities or exacerbating the concomitant infection made the patient’s gout more difficult to quell. This added to the difficulty to manage this patient’s bacteremia. Adequate education for the patient and caregiver on appropriate wound care and the use of ULTs is of the utmost importance for longitudinal care.

## References

[1] Singh G, Lingala B, Mithal A (2019). Gout and hyperuricaemia in the USA: prevalence and trends. Rheumatology (Oxford).

[2] Al-Ashkar F (2022). Gout and calcium pyrophosphate deposition disease. https://www.clevelandclinicmeded.com/medicalpubs/diseasemanagement/rheumatology/gout-and-pseudogout/#gout.%20Published%20June.

[3] Spaetgens B, de Vries F, Driessen JH (2017). Risk of infections in patients with gout: a population-based cohort study. Sci Rep.

[4] Lim SY, Lu N, Choi HK (2015). Septic arthritis in gout patients: a population-based cohort study. Rheumatology (Oxford).

[5] Chakraborty RK, Burns B (2022). Systemic Inflammatory Response Syndrome. https://www.ncbi.nlm.nih.gov/books/NBK547669/.

[6] Singh JA, Gaffo A (2020). Gout epidemiology and comorbidities. Semin Arthritis Rheum.

[7] Sivera F, Andrés M, Carmona L (2014). Multinational evidence-based recommendations for the diagnosis and management of gout: integrating systematic literature review and expert opinion of a broad panel of rheumatologists in the 3e initiative. Ann Rheum Dis.

[8] Janssen CA, Oude Voshaar MA, Vonkeman HE (2019). Anakinra for the treatment of acute gout flares: a randomized, double-blind, placebo-controlled, active-comparator, non-inferiority trial. Rheumatology (Oxford).

[9] Falidas E, Rallis E, Bournia VK, Mathioulakis S, Pavlakis E, Villias C (2011). Multiarticular chronic tophaceous gout with severe and multiple ulcerations: a case report. J Med Case Rep.

[10] Thueringer JT, Doll NK, Gertner E (2015). Anakinra for the treatment of acute severe gout in critically ill patients. Semin Arthritis Rheum.

